# Starvation Induced Cell Death in Autophagy-Defective Yeast Mutants Is Caused by Mitochondria Dysfunction

**DOI:** 10.1371/journal.pone.0017412

**Published:** 2011-02-25

**Authors:** Sho W. Suzuki, Jun Onodera, Yoshinori Ohsumi

**Affiliations:** 1 Frontier Research Center, Tokyo Institute of Technology, Yokohama, Japan; 2 Graduate School of Bioscience, Tokyo Institute of Technology, Yokohama, Japan; 3 Department of Cell Biology, Division of Molecular Cell Biology, National Institute for Basic Biology, Okazaki, Japan; Roswell Park Cancer Institute, United States of America

## Abstract

Autophagy is a highly-conserved cellular degradation and recycling system that is essential for cell survival during nutrient starvation. The loss of viability had been used as an initial screen to identify autophagy-defective (*atg*) mutants of the yeast *Saccharomyces cerevisiae*, but the mechanism of cell death in these mutants has remained unclear. When cells grown in a rich medium were transferred to a synthetic nitrogen starvation media, secreted metabolites lowered the extracellular pH below 3.0 and autophagy-defective mutants mostly died. We found that buffering of the starvation medium dramatically restored the viability of *atg* mutants. In response to starvation, wild-type (WT) cells were able to upregulate components of the respiratory pathway and ROS (reactive oxygen species) scavenging enzymes, but *atg* mutants lacked this synthetic capacity. Consequently, autophagy-defective mutants accumulated the high level of ROS, leading to deficient respiratory function, resulting in the loss of mitochondria DNA (mtDNA). We also showed that mtDNA deficient cells are subject to cell death under low pH starvation conditions. Taken together, under starvation conditions non-selective autophagy, rather than mitophagy, plays an essential role in preventing ROS accumulation, and thus in maintaining mitochondria function. The failure of response to starvation is the major cause of cell death in *atg* mutants.

## Introduction

Unlike the ubiquitin-proteasome system that requires strict recognition of targets [1], autophagy is a non-selective degradation system induced under starvation conditions that mediates the recycling of cytoplasmic components to supply amino acids [2], [3]. Despite normal gene transcription, autophagy-deficient yeast cells have impaired protein translation during starvation due to inadequate supplies of amino acids [4]. Autophagy is critical for starvation-induced differentiation or development of yeast, nematodes, flies, and mice [5]. Accumulating data indicate that autophagy plays important roles in maintaining cellular homeostasis, and is relevant to several diseases [6], [7].

Mitochondria generate ATP through respiratory chain activity, but reactive oxygen species (ROS) are generated as by-products of cellular respiration. ROS induce damages to membrane, DNA, protein, and organelles, therefore mechanisms regulating the function and quantity of mitochondria are essential for eukaryotic cell function. Autophagy contributes for mitochondria maintenance by their clearance [8], and this process is mediated by selective type of autophagy termed mitophagy [9]–[12].

In *S. cerevisiae*, autophagy-defective (*atg*) mutants exhibit reduced viability during nitrogen starvation [13], but the mechanism of cell death has been unclear. Here, we show that non-selective autophagy is necessary to maintain the synthesis of respiratory chain components and ROS scavengers. In autophagy-deficient cells, mitochondrial dysfunction induced during starvation leads to impaired cell viability.

## Results

### pH buffering allows *atg* mutants to survive nitrogen starvation

**Figure 1:**
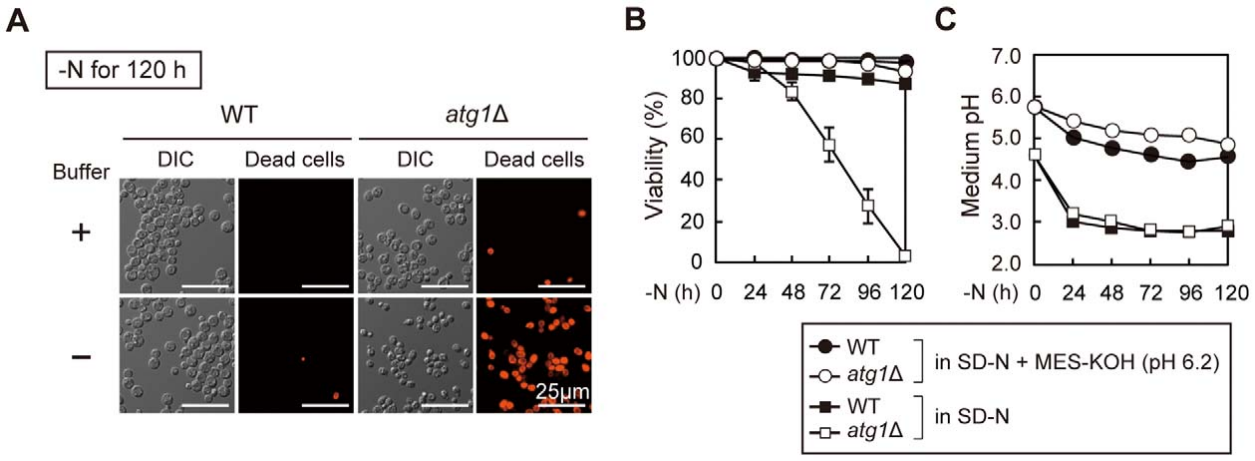
The viability of *atg* mutants in buffered starvation medium. (A) WT and *atg1*Δ cells grown in YEPD medium were transferred to SD-N or SD-N +50 mM MES-KOH (pH 6.2) medium for 120 hours, and dead cells stained by phloxine B were observed by fluorescent microscopy. Scale bar, 25 µm. (B–C) WT and *atg1*Δ cells grown nitrogen starved as (A) for the indicated times. Cell viability (B) and medium pH (C) were examined by phloxine B staining and pH meter, respectively. These data represent the average of three independent experiments and bars indicate standard deviations.

**Figure 2:**
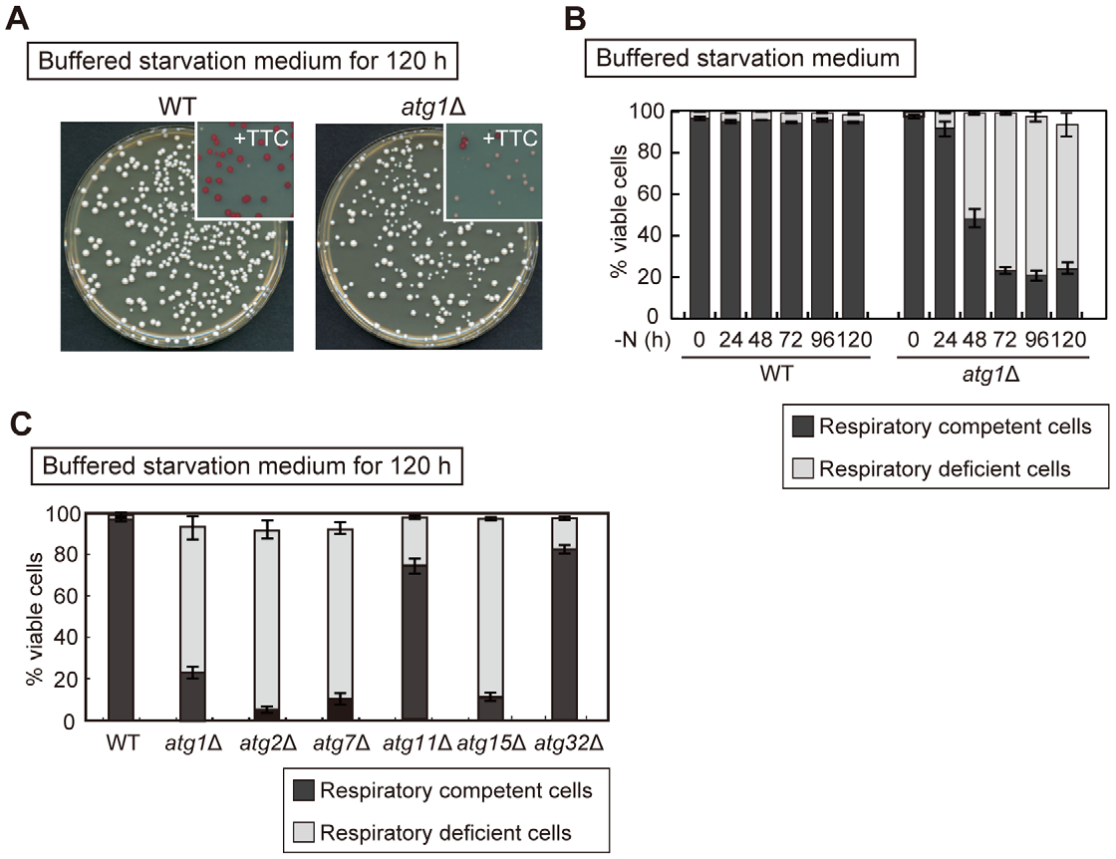
Loss of respiratory function in *atg* mutants during nitrogen starvation. (A) WT and *atg1*Δ cells grown in YEPD medium were transferred to SD-N +50 mM MES-KOH (pH 6.2). After 120 hours, cultures were diluted 5.0×10^5^ fold and plated onto YEPD agar. The plates were incubated at 30°C for four days. Insets indicate the plate overlaid with TTC agar to examine the respiratory competency of formed colonies. (B) WT, and *atg1*Δ cells were nitrogen-starved as (A). Cell viability and respiratory competency was determined by phloxine B staining and TTC overlay technique as (A), respectively. The black and gray areas indicate the percentage of viable cells that are respiratory competent or respiratory deficient, respectively. (C) WT, *atg1*Δ, *atg2*Δ, *atg7*Δ, *atg11*Δ, *atg15*Δ, and *atg32*Δ cells were nitrogen-starved as (A). Cell viability and respiratory competency was determined by phloxine B staining and TTC overlay technique as (A), respectively. The black and gray areas indicate the percentage of viable cells that are respiratory competent or respiratory deficient, respectively. These data represent the average of three independent experiments and bars indicate standard deviations.

Autophagy is required for cell survival under conditions of nitrogen starvation in yeast [13], and to better understand the cellular events occurring during starvation we examined wild-type (WT) or autophagy-deficient *atg1*Δ cells during nitrogen starvation. Logarithmically growing *atg1*Δ cells were transferred to a synthetic medium without nitrogen sources (SD-N, referred to as non-buffered starvation medium), and their viability was monitored for five days using phloxine B, a reagent that stains dead cells. As previously reported, *atg1* mutant cells exhibited decreased viability after two days of starvation, and almost completely *atg1*Δ cells died after five days (3.0% of survival cells). In contrast, 87.2% of WT cells were viable at that time (Figure 1A and 1B). The loss of viability was used as an initial screen to obtain *atg* mutants [13], but the cause of cell death in autophagy-defective mutants remained unclear. During these cultures, due to secreted metabolites, the pH of both the WT and *atg1*Δ starvation media decreased and reached a plateau below pH 3.0 (Figure 1C). We hypothesized that maintaining a neural pH could promote cell survival, and when we added MES-KOH (pH 6.2) to the starvation medium (hereafter referred to as buffered starvation medium), the extracellular pH was maintained near pH 5.0 after five days (Figure 1C). Under these conditions, 93.4% of *atg1*Δ cells remained viable after 120 hours of nitrogen starvation (Figure 1A and 1B), and similar rates of viability were seen for other *atg* mutants (*atg2*Δ, *atg7*Δ, *atg11*Δ, *atg15*Δ) (Figure 2C). To confirm that pH buffering was responsible for these observations, we used MES-NaOH (pH 6.2) as buffer, and it also maintained the viability of the autophagy-defective mutants (Figure S1). Additionally, MES-KOH (pH 5.0) slightly restored cell viability, but to a lower extent than MES-KOH (pH 6.2) (Figure S1). Cell viability in the non-buffered starvation medium containing KCl such that concentration of potassium ion is equivalent to that in MES-KOH (pH 6.2) buffering media did not improve (Figure S1). Thus, under conditions of nitrogen starvation, autophagy mutants are very sensitive to the extracellular pH, and more acidic medium leads to greater cell death.

### Autophagy is required for the maintenance of respiratory function during starvation

Buffered starvation medium dramatically improves cell viability of *atg* mutants under long nitrogen starvation (Figure 1A and 1B). We determined the cell viability also by colony forming ability on YEPD plates and obtained similar results with phloxine B staining (data not shown). In the course of this experiment, we noticed that *atg1*Δ cells formed small colonies at high rate (Figure 2A). In many cases in yeast, small colonies reflect impaired respiratory function, so we examined the respiratory competency of the cells under starvation conditions using 2,3,5-triphenyltetrazolium chloride (TTC), which stains respiratory competent colonies red [14]. Most of the small colonies derived from *atg1* mutants were not colored (Figure 2A), implying that autophagy-deficient cells lost their respiratory activity. To better understand the kinetics of this observation, we monitored the respiratory competency of *atg1*Δ cells by TTC staining. After 24 hours of nitrogen starvation, *atg1*Δ cells gradually lost their respiratory activity, and by 72 hours 75.5% of the *atg1*Δ cells lost their respiratory function, although only 5.0% of WT cells showed respiratory defect (Figure 2B). Other autophagy-defective mutants (*atg2*Δ, *atg7*Δ) that are unable to form autophagosomes lost respiratory function with similar kinetics (Figure 2C).

### Autophagy plays a role to retain mtDNA during starvation

**Figure 3:**
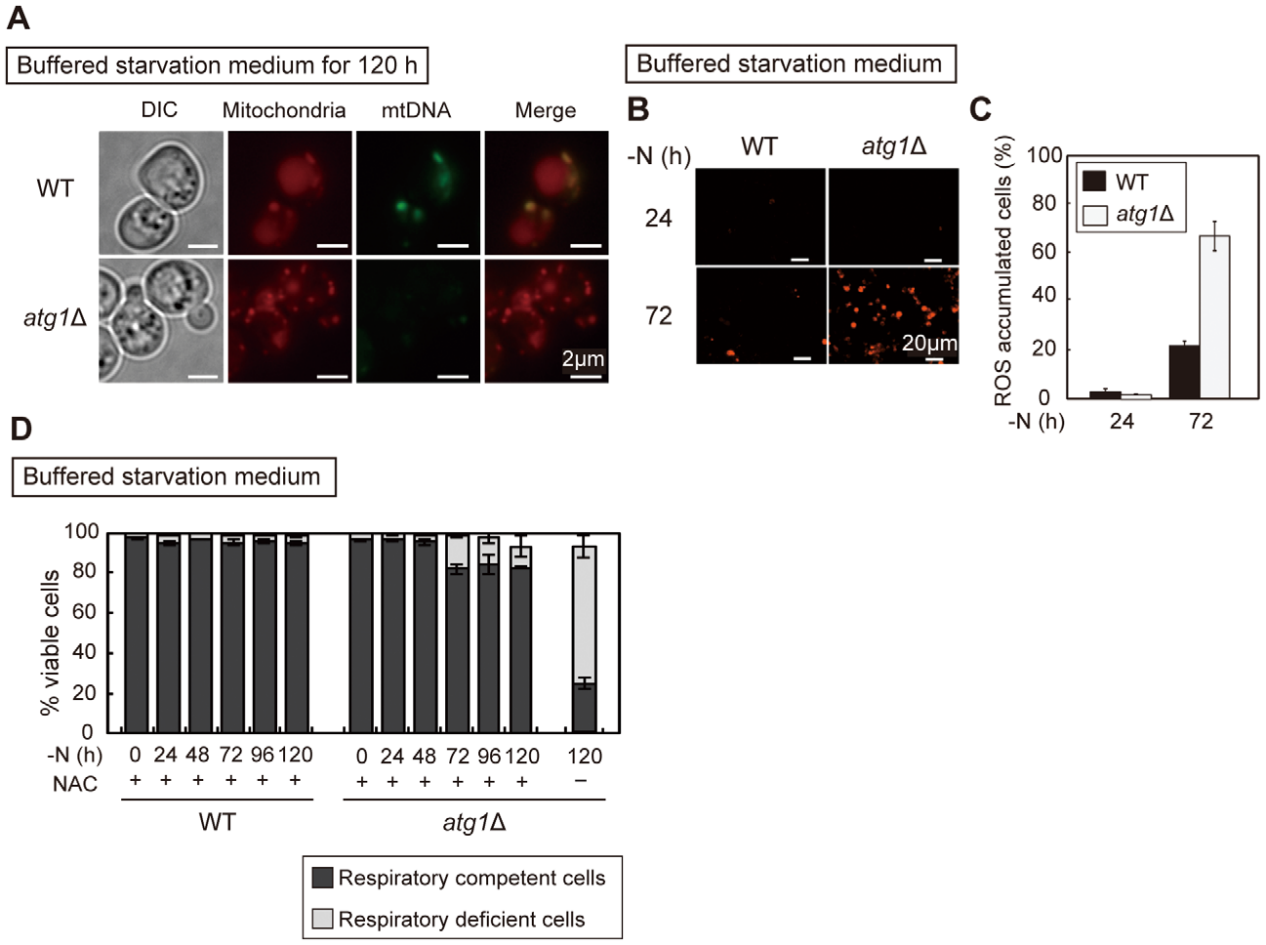
Mitochondrial defect in *atg* mutants. (A) WT and *atg1*Δ cells expressing mitochondria targeted mCherry cultured in SD-N +50 mM MES-KOH (pH 6.2) medium for 120 hours were observed by fluorescent microscopy. Mitochondrial DNA was stained with SYBR green I, and mitochondria were visualized by mitochondria targeted mCherry. Scale bar, 2 µm. (B–C) WT and *atg1*Δ cells were transferred to SD-N +50 mM MES-KOH (pH 6.2) medium for the indicated time. ROS accumulation was detected by DHE staining (B). Each photo contains about 200 cells. Scale bar, 20 µm. (C) shows quantification of ROS accumulated cells (n>200 cells). (D) WT and *atg1*Δ cells were transferred to SD-N +50 mM MES-KOH (pH 6.2) with 10 mM NAC for the indicated time. Cell viability was determined by phloxine B staining. Cells from these cultures were plated on YEPD agar and overlaid with TTC agar to examine respiratory competency. The black and gray areas indicate the percentage of viable cells that are respiratory competent or respiratory deficient, respectively. These data represent the average of three independent experiments and bars indicate standard deviations.

We next analyzed the mechanism by which autophagy-deficient cells lose their respiratory function. We examined if mitochondria retained mtDNA (mitochondria DNA) in *atg* mutant cells. Mitochondria were visualized by mitochondria targeted mCherry, and mtDNA was labeled with SYBR green. Both WT and *atg1*Δ cells contained mtDNA until 24 hours of nitrogen starvation (Figure S2). After 120 hours of starvation, the mitochondria in WT cells retained mtDNA, but nearly all of the *atg1*Δ cell lost their mtDNA (Figure 3A). We confirmed that the small colonies derived from culture grown in buffered starvation medium were comprised of rho^0^ cells, mitochondria DNA deficient cells, by DAPI staining (Figure S3), which stains both nuclear DNA and mtDNA. These results suggest that autophagy-defective mutants become rho^0^ cells during nitrogen starvation.

### ROS are a major factor contributing to the loss of respiratory function

ROS induce mutations in mtDNA, leading to the loss of respiratory function in mice [15]. Additionally, autophagy-deficient yeast cells accumulate ROS to a higher extent than WT cells during stationary phase [16]. We hypothesized that accumulated ROS cause loss of mtDNA in *atg* mutant cells, and investigated ROS accumulation during nitrogen starvation using dihydroethidium (DHE). When both WT and *atg1*Δ cells were transferred to buffered starvation medium, ROS levels transiently increased. After 72 hours, the *atg1*Δ cells (67.0% ROS accumulated cells) accumulated more ROS than WT cells (21.2% ROS accumulated cells) (Figure 3B and 3C). When the antioxidant N-acetylcysteine (NAC) was added to the starvation medium, ROS accumulation was suppressed, and the loss of respiratory function by *atg1*Δ cells was abrogated (Figure 3D). These results suggest that autophagy is required to prevent excessive ROS accumulation during starvation, and ROS accumulation impairs respiratory function in autophagy-defective mutants.

### Inability to increase the expression of respiratory components and ROS scavenger enzyme led ROS accumulation

**Figure 4:**
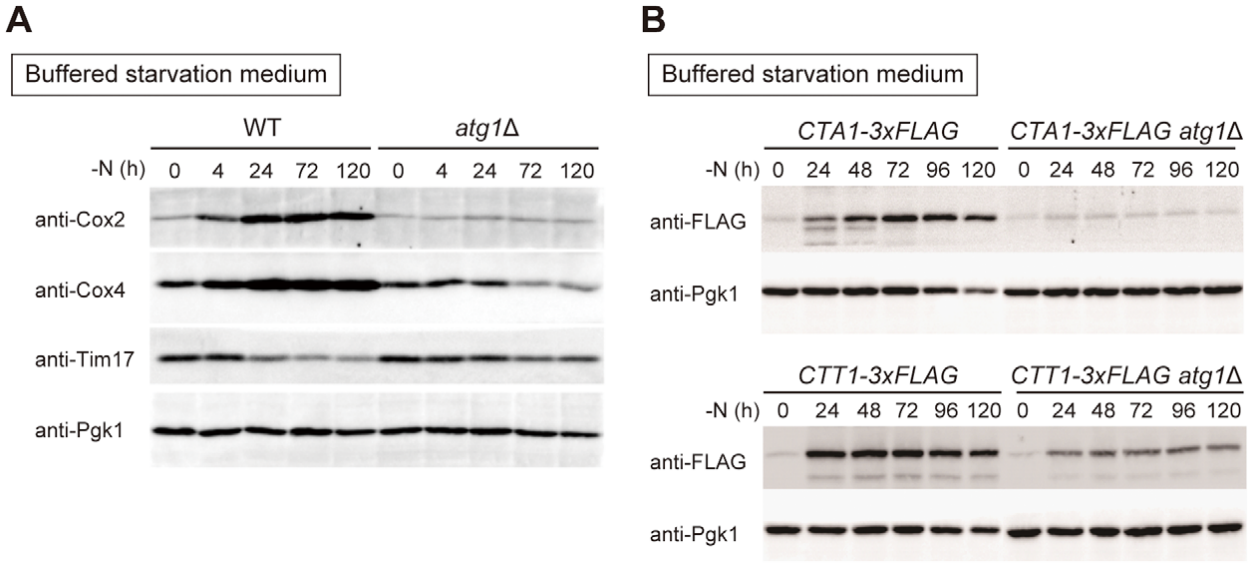
Altered expression of respiratory components and ROS scavengers by *atg* mutant cells. (A) WT and *atg1*Δ cells were transferred to SD-N +50 mM MES-KOH (pH 6.2) medium for the indicated time. Lysates were prepared using the alkaline-trichloroacetic acid method and subjected to immunoprecipitation with anti-Cox2, anti-Cox4, anti-Tim17 and anti-Pgk1. Pgk1 was used as loading control. (B) WT and *atg1*Δ cells expressing Cta1-3×FLAG or Ctt1-3×FLAG were nitrogen-starved as in (A) for the indicated time. Cell lysates were prepared as (A) and subjected to immune precipitation with anti-FLAG and anti-Pgk1. Pgk1 was used as loading control.

It is known that the electron transport system is the major source of ROS production during respiratory growth [15], [17], [18], so we examined the accumulation of ROS during starvation in rho^0^ *atg1*Δ cells. Compared to *atg1*Δ cells (67.0% ROS accumulated cells), there was little accumulation of ROS in rho^0^ *atg1*Δ cells (3.0% of ROS accumulated cells) (Figure S4A and S4B), implying that respiration is the major source of ROS in *atg* mutants during nitrogen starvation. Alterations in components of the respiratory chain that affect reaction efficiency promote electron transport to oxygen and the generation of ROS [15]. To probe the composition of the respiratory pathway, we examined the expression of Cox4, a subunit of cytochrome C oxidase encoded in the nuclear genome, and Cox2, another cytochrome C oxidase component encoded in the mtDNA [19]. Expression of both Cox2 and Cox4 was increased in WT cells in response to nitrogen starvation, but no significant increase in Cox2 or Cox4 expression occurred in *atg1*Δ cells (Figure 4A). It is likely that ROS generation is more pronounced in *atg* mutants because respiratory chain function deviates from optimum conditions. On the other hand, the amount of Tim17, a subunit of the translocase of the mitochondrial inner membrane, slightly decreased in both WT and *atg1*Δ cells during starvation (Figure 4A), suggesting that mitochondrial protein composition changes during starvation and expression of these newly synthesized proteins depends on intact autophagy.

Several pathways have evolved to scavenge ROS, and we examined the expression of Cta1 and Ctt1, both of which catalyze the decomposition of ROS to water and oxygen [20], [21]. Expression of both Cta1 and Ctt1 was immediately increased upon shifting WT cells to nitrogen starvation medium, but expression of these scavenger proteins was not relatively increased in *atg1*Δ cells upon starvation (Figure 4B). Thus, the inability to increase the expression of respiratory chain components and ROS scavengers likely leads to the accumulation of ROS in autophagy-defective cells.

### Vacuolar degradation by non-selective autophagy, rather than selective autophagy, is important for the maintenance of respiratory function

Autophagy is involved in not only the maintenance of amino acid levels but also cellular homeostasis by sequestration of aberrant organelles, therefore we assessed another possibility that sequestration of dysfunctional organelles by autophagy contributes for the maintenance of respiratory functions. Mitochondria is selectively degraded in the vacuoles via autophagy termed mitophagy [9], [10], and Atg32, a mitochondria-anchored protein, is essential for this process [11], [12]. However, a substantial fraction of *atg32*Δ cells retained their respiratory function after 120 hours of nitrogen starvation (Figure 2C), indicating that mitophagy is not significantly involved in the maintenance of respiratory function. Other selective types of autophagy such as the Cvt (cytoplasm to vacuole targeting) pathway and pexophagy as well as mitophagy require the Atg11 protein [22], but *atg11*Δ cells did not show respiratory defect. In contrast, *atg15*Δ cells, which cannot perform the intravacuolar disintegration of autophagic bodies [23], inner membrane-bound structures of autophagosomes, exhibited significant respiratory defect (Figure 2C). Taken together, vacuolar degradation of cytoplasmic components by non-selective autophagy, rather than selective autophagy, is crucial for the maintenance of respiratory function during nitrogen starvation.

### Defective respiration leads to cell death in *atg* mutants

**Figure 5:**
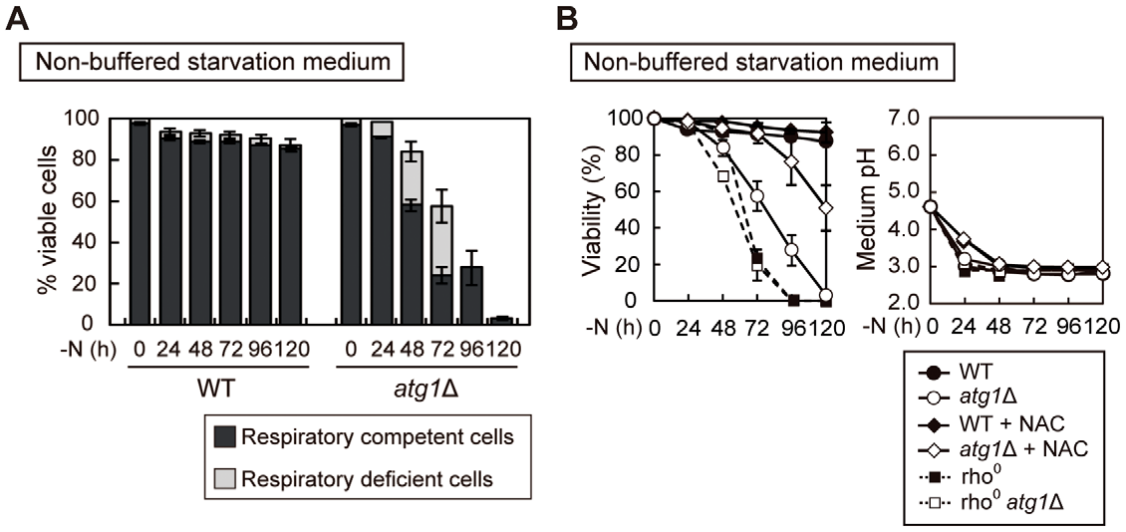
Viability of respiratory deficient cells during nitrogen starvation. (A) WT and *atg1*Δ cells were transferred to non-buffered SD-N medium for the indicated time. Cell viability was determined by phloxine B staining. Nitrogen-starved cells were plated onto YEPD agar and overlaid with TTC agar to examine the respiratory competency of formed colonies. The black and gray areas indicate the percentage of viable cells that are respiratory competent or respiratory deficient, respectively. (B) WT, *atg1*Δ, rho^0^, and rho^0^ *atg1*Δ cells grown in YEPD medium were transferred to SD-N with or without 10 mM NAC for the indicated time. In the presence of NAC, medium pH was adjusted by using KOH. Cell viability and medium pH were examined by phloxine B staining and pH meter, respectively. These data represent the average of three independent experiments and bars indicate standard deviations.

In *atg* mutants, the time course of the appearance of the respiratory-deficient cells in buffered starvation medium temporally corresponded to the loss of cell viability seen in non-buffered starvation medium. We monitored the respiratory activity of autophagy-defective mutants in non-buffered starvation medium, and after 96 hours of starvation nearly all of the surviving *atg1*Δ cells exhibited respiratory activity (Figure 5A), indicating that respiratory-deficient cells were selectively eliminated.

We next determined whether the cell death observed in non-buffered starvation medium was due to the loss of respiratory function. The viability of respiratory-defective rho^0^ cells, which completely lack mtDNA, was monitored in non-buffered starvation medium (Figure 5B). After 72 hours of nitrogen starvation 57.6% of *atg1*Δ cells were viable, but only 22.8% of rho^0^ cells survived, though the pH of the media of both cultures decreased at the same rate (Figure 5B). We confirmed that autophagy is normally induced in rho^0^ cells (Figure S5A). When starved in the buffered starvation media, both rho^0^ and rho^0^ *atg1*Δ cells showed high viability (Figure S5B). In the presence of NAC, lethality of *atg1*Δ cells was rescued in non-buffered starvation medium (Figure 5B). NAC raises the medium pH slightly, but the extent of restored viability seems not to be explained by this buffering effect (See Figure S1). Taken together, these data suggest that respiratory deficient cells are exquisitely sensitive to low pH under conditions of nitrogen starvation compared with respiratory competent cells. Our data indicate that accumulated ROS in *atg* mutants impaired respiratory function, which renders cells sensitive to the low pH environment, leading to greater cell death in non-buffered starvation media.

## Discussion

In this study, we showed links between impaired autophagy, mitochondrial injury and dysfunction, and, ultimately, cell death under conditions of nitrogen starvation.

We previously reported that autophagy-defective mutants cannot maintain the amino acid pool needed for protein synthesis, and this impairs the ability of cells to upregulate the expression of starvation-induced genes [4]. In the present study, we found proteins involved in cellular respiration encoded both chromosomally (i.e. Cox4) and by the mtDNA (i.e. Cox2) upregulated under starvation conditions (Figure 4A). The ROS scavenger proteins Cta1 and Ctt1 also augmented under such conditions and autophagy is required for upregulation of these starvation-induced proteins (Figure 4B), consequently *atg* mutants show the increased production of ROS and reduced ROS scavenging activity (Figure 3B and 3C), likely leading mitochondria dysfunction (Figure 2B). Consistently, treatment of cells with the antioxidant NAC suppressed the loss of respiratory activity and restored cell viability during starvation in *atg* mutants (Figure 3D and 5B). In this context, autophagy is essential for synthesis of minimal essential proteins to maintain mitochondrial function during nitrogen starvation.

Autophagy could contribute to the elimination of damaged mitochondria and, in this way, prevent the accumulation of ROS [8]. Mitochondria are non-selectively incorporated into autophagosomes during starvation [24], but they are also selectively degraded via mitophagy, in which Atg32 is requisite [11], [12]. However, *atg32*Δ cells showed nearly normal respiratory activity (Figure 2C), indicating that selective mitochondria elimination is not responsible for the observed respiratory defects caused in *atg* mutants under starvation conditions. On the other hand, cells lacking *ATG15*, which can form autophagosomes but not degrade the contents [23], exhibited respiratory deficient cells (Figure 2C). It is likely that sequestration of cytoplasmic components is not sufficient to retain respiratory function, but impaired autophagic degradation of cytoplasmic components is responsible for the maintenance of respiratory function.

In this study, we proposed that supply of amino acids via autophagy is important for the maintenance of respiratory function under starvation conditions. After 120 hours nitrogen starvation *atg* mutants exhibit respiratory defects dramatically (WT: 3.8%, *atg1*Δ: 75.5%) (Figure 2B). Recently, Zhang et al. presented that stationary phase *atg* mutant cells shows higher spontaneous petite frequencies up to 6% than about 3% in WT cells [16]. The total cellular amino acid levels in autophagy deficient cells drastically decrease under starvation conditions compared to those under nutrient rich conditions [4]. It is likely that amino acid pool in nitrogen starved *atg* mutant is smaller than that in stationary phase *atg* mutant. It might account for the difference between rates of respiratory deficient cells of Zhang's result and our result.

The mechanisms by which excess ROS can induce the loss of mtDNA in autophagy-defective mutants remain unclear. It is known that the loss of mtDNA is caused by inhibition of mtDNA replication during cell division [25]. Defect in mtDNA replication for several generations decreases inherited mtDNA number, and finally cells become rho^0^ mutants. However, nitrogen-starved cells are arrested at the first cell cycle [24], therefore a different unknown mechanism independent of cell proliferation must be involved.

We suggested that the maintenance of mitochondria function is important for cell survival during cell starvation. Recently, the relationship between autophagy and chronological life span had been discussed [8], [26], and Fabrizio et al. presented that deletion of genes involved in autophagy or mitochondria functions shorten life span in *Saccharomyces cerevisiae* [27]. Matecic et al. reported that raising medium pH to 6.0 also induces the extension of chronological life span in *atg* mutants [28]. Thus, essential factors for long-term survival and adaptation to starvation conditions have common features, and neutralizing medium suppress short life span or starvation induced cell death in *atg* mutants. Shorten chronological life span in autophagy-defective mutants may be at least partly attributed to mitochondria dysfunction.

We revealed that buffered starvation medium allows autophagy deficient cells to survive five days of nitrogen starvation (Figure 1A and 1B). It allows us to investigate autophagy during long nitrogen starvation. Until now, we observed that lipid droplets are transported to the vacuole in an autophagy dependent manner under prolonged starvation (manuscript in preparation). Thus, buffered starvation conditions will be useful to elucidate novel autophagic processes.

Autophagy has been implicated in a variety of essential cellular functions, and we have established an important role for autophagy in maintaining mitochondrial function by supporting essential protein synthesis, and mitochondria dysfunction is the major cause of starvation-induced cell death in autophagy-defective mutants. Further experiments in yeast will provide mechanistic insights into physiological roles of autophagy.

## Materials and Methods

### Yeast Strains and Media

The yeast strains used in this study are listed in Table S1. The mutant strains were generated using standard genetic and molecular biology techniques. To investigate nitrogen-starved cells, cells grown in rich medium (YEPD medium: 1% yeast extract, 2% peptone, and 2% glucose) were shifted to nitrogen starvation medium (SD-N medium: 0.17% yeast nitrogen base without amino acids and ammonium sulfate and 2% glucose). Yeast cells were grown in YEPD medium to an OD_600_ of 1.4 at 30°C. These cells were washed with distilled water, resuspended in SD-N medium and cultured for the indicated times at 30°C. The rho^0^ derivative was obtained by ethidium bromide treatment as described [25].

### Microscopy and live cell imaging

Cells were observed using an inverted microscope (IX71 or IX81; Olympus) equipped with differential interference contrast optics, epifluorescence capabilities, 60× (UPlanApo 60×, NA: 1.40; Olympus), 100× (UPlanApo 100×, NA: 1.35; Olympus) or 150× (UApo N 150×, NA: 1.45; Olympus) objective lenses, a EM-CCD camera (ImagEM Enhanced; HAMAMATSU PHOTONICS) or a monochrome CCD camera (CoolSNAP HQ; Roper) or, and filter sets for mCherry, DHE staining, DAPI staining and SYBR-Green I staining. Images were captured using image acquisition and analysis software (AQUACOSMOS or MetaMorph 7.0r4; Molecular Devices).

### Immunoblotting

Samples corresponding to 5.0×10^−2^ OD_600_ units of cells were separated by SDS-PAGE followed by the western blotting and immunodecoration. After treatment with enhanced chemiluminescence reagents, proteins were detected using a luminescent image analyzer (LAS-4000 mini; Fujifilm). Anti-Cox2, Anti-Cox4, and Anti-Tim17 were gifts from Dr. Endo, Nagoya University, Japan. To detect GFP, Flag, and Pgk1, anti-GFP antiserum (Roche), Anti-Flag antiserum (Sigma), and Anti-Pgk1 (Invitrogen) were used, respectively.

### Determination of cell viability

Cells viability was determined by phloxine B (final concentration 5 µg/ml) staining. Cell cultures were examined by fluorescence microscopy with a blue filter. Brightly fluorescent cells were counted as dead cells. About 200 cells were counted at each time point.

### Measurement of medium pH

Medium pH was measured using a pH electrode/meter (Horiba).

### Triphenyltetrazolium chloride staining

Respiratory competency was determined by the TTC staining method. TTC agar overlay was carried out as described [14].

### Detection of reactive oxygen species (ROS)

Reactive oxygen species were detected by DHE (dihydroethidium; final concentration 5 µg/ml) staining. Cells were incubated at 30°C for 20 min, washed with distilled water, resuspended in distilled water and examined by fluorescence microscopy with a blue filter.

## Supporting Information

Figure S1 **The viability of** ***atg1*** **mutants in various starvation medium.** *atg1*Δ cells grown in YEPD medium were transferred to the indicated starvation medium for the indicated time. Cell viability and medium pH were examined by phloxine B staining and pH meter, respectively. (○); *atg1*Δ cells in SD-N +50 mM MES-KOH (pH 6.2), (⧫); *atg1*Δ cells in SD-N +50 mM MES-NaOH (pH 6.2), (◊); *atg1*Δ cells in SD-N +50 mM MES-KOH (pH 5.0), (▪); *atg1*Δ cells in SD-N added 5 mM KCl to adjust at a same potassium concentration with MES-KOH (pH 6.2). These data represent the average of three independent experiments and bars indicate standard deviations.
(TIF)

Figure S2 **Mitochondria DNA in nitrogen-starved** ***atg*** **mutants.** WT and *atg1*Δ cells expressing mitochondria targeted mCherry cultured in SD-N +50 mM MES-KOH (pH 6.2) medium for 24 hours were observed by fluorescent microscopy. Mitochondrial DNA was stained with SYBR green I, and mitochondria were visualized by mitochondrial targeting mCherry. Scale bar, 5 µm.
(TIF)

Figure S3 **DNA in cells derived from small size of colony.** *atg1*Δ cells grown in YEPD medium were transferred to SD-N +50 mM MES-KOH (pH 6.2). After 120 hours, cultures were diluted 5.0×10^5^ fold and plated onto YEPD agar. The plates were incubated at 30°C for four days. Cells derived from large or small size of colony culture in YEPD medium. Yeast cells were grown to an OD_600_ of 0.6 at 30°C and 2.5 µg/ml DAPI was added to the medium to detect both nuclear DNA and mtDNA. Before subjecting to microscopy cells were washed with distilled water, resuspended in distilled water and observed by fluorescence microscopy. Scale bar, 5 µm.
(TIF)

Figure S4 **ROS generation in respiratory defective cells.** (A–B) WT, *atg1*Δ, rho^0^, and rho^0^ *atg1*Δ cells were transferred to SD-N +50 mM MES-KOH (pH 6.2) medium for 72 hours. ROS accumulation was detected by DHE staining (A). Each photo contains about 200 cells. Scale bar, 20 µm. (B) Quantification of ROS accumulated cells (n>200 cells). This data represents the average of three independent experiments and bars indicate standard deviations.
(TIF)

Figure S5 **Respiratory deficient cells during nitrogen starvation.** (A) WT, *atg1*Δ, rho^0^, and rho^0^ *atg1*Δ cells expressing GFP-Atg8 were transferred to SD-N +50 mM MES-KOH (pH 6.2) medium for the indicated time. During autophagy process, GFP-Atg8 (depicted by arrow) is delivered to the vacuole, and hydrolyzed to generate free GFP (depicted by arrowhead). Generation of free GFP indicates transport of the marker to the vacuole. Lysates were prepared using a Multi-Beads Shocker (model MB601NIHS, Yasui Kikai Co. Osaka, Japan) and subjected to immunoprecipitation with anti-GFP and anti-Pgk1. Pgk1 was used as loading control. (B) WT, *atg1*Δ, rho^0^, and rho^0^ *atg1*Δ cells were transferred to SD-N or SD-N +50 mM MES-KOH (pH 6.2). Cell viability was examined by phloxine B. This data represents the average of three independent experiments and bars indicate standard deviations.
(TIF)

Table S1 **Yeast strains used in this study.**
(PDF)
