# Supplementary figures and images for: Starvation Induced Cell Death in Autophagy-Defective Yeast Mutants Is Caused by Mitochondria Dysfunction
Source: PLoS One. 2011 Feb 25;6(2):e17412. doi: 10.1371/journal.pone.0017412 (PMC3045454; doi:10.1371/journal.pone.0017412)

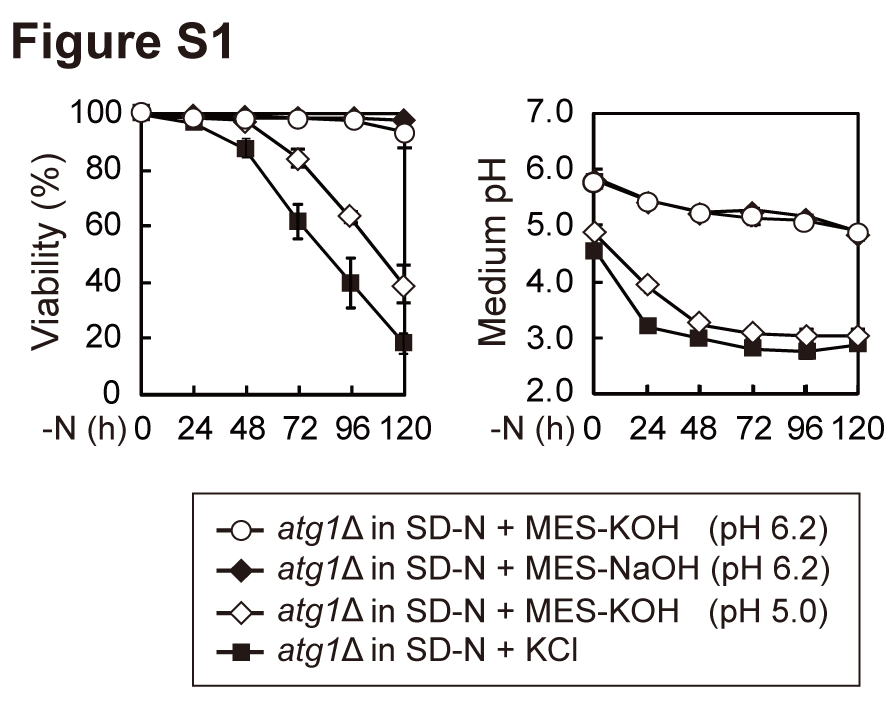

Supplement: Figure S1 — The viability of atg1 mutants in various starvation medium. atg1Δ cells grown in YEPD medium were transferred to the indicated starvation medium for the indicated time. Cell viability and medium pH were examined by phloxine B staining and pH meter, respectively. (○); atg1Δ cells in SD-N +50 mM MES-KOH (pH 6.2), (⧫); atg1Δ cells in SD-N +50 mM MES-NaOH (pH 6.2), (◊); atg1Δ cells in SD-N +50 mM MES-KOH (pH 5.0), (▪); atg1Δ cells in SD-N added 5 mM KCl to adjust at a same potassium concentration with MES-KOH (pH 6.2). These data represent the average of three independent experiments and bars indicate standard deviations. (TIF) [file pone.0017412.s001.tif]

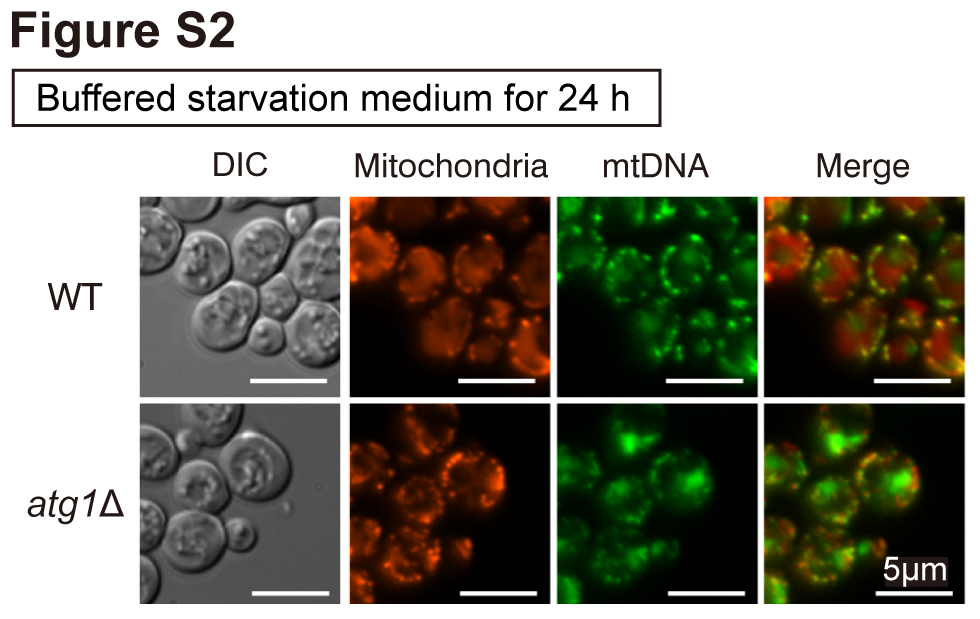

Supplement: Figure S2 — Mitochondria DNA in nitrogen-starved atg mutants. WT and atg1Δ cells expressing mitochondria targeted mCherry cultured in SD-N +50 mM MES-KOH (pH 6.2) medium for 24 hours were observed by fluorescent microscopy. Mitochondrial DNA was stained with SYBR green I, and mitochondria were visualized by mitochondrial targeting mCherry. Scale bar, 5 µm. (TIF) [file pone.0017412.s002.tif]

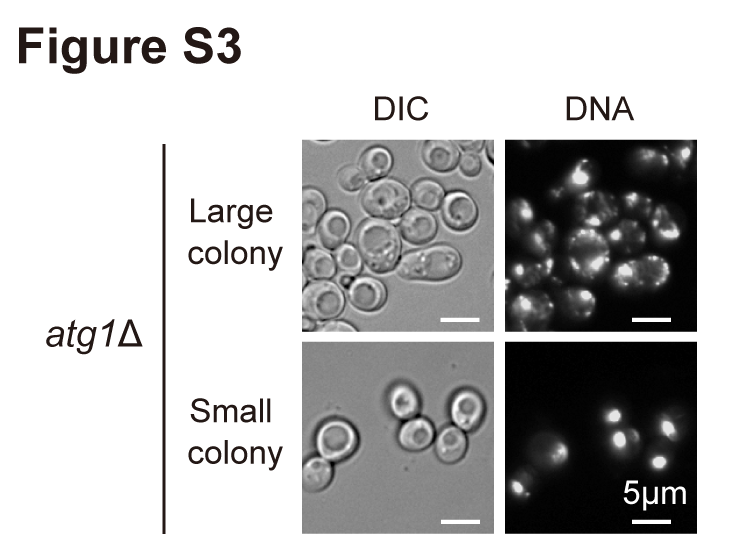

Supplement: Figure S3 — DNA in cells derived from small size of colony. atg1Δ cells grown in YEPD medium were transferred to SD-N +50 mM MES-KOH (pH 6.2). After 120 hours, cultures were diluted 5.0×10^5^ fold and plated onto YEPD agar. The plates were incubated at 30°C for four days. Cells derived from large or small size of colony culture in YEPD medium. Yeast cells were grown to an OD600 of 0.6 at 30°C and 2.5 µg/ml DAPI was added to the medium to detect both nuclear DNA and mtDNA. Before subjecting to microscopy cells were washed with distilled water, resuspended in distilled water and observed by fluorescence microscopy. Scale bar, 5 µm. (TIF) [file pone.0017412.s003.tif]

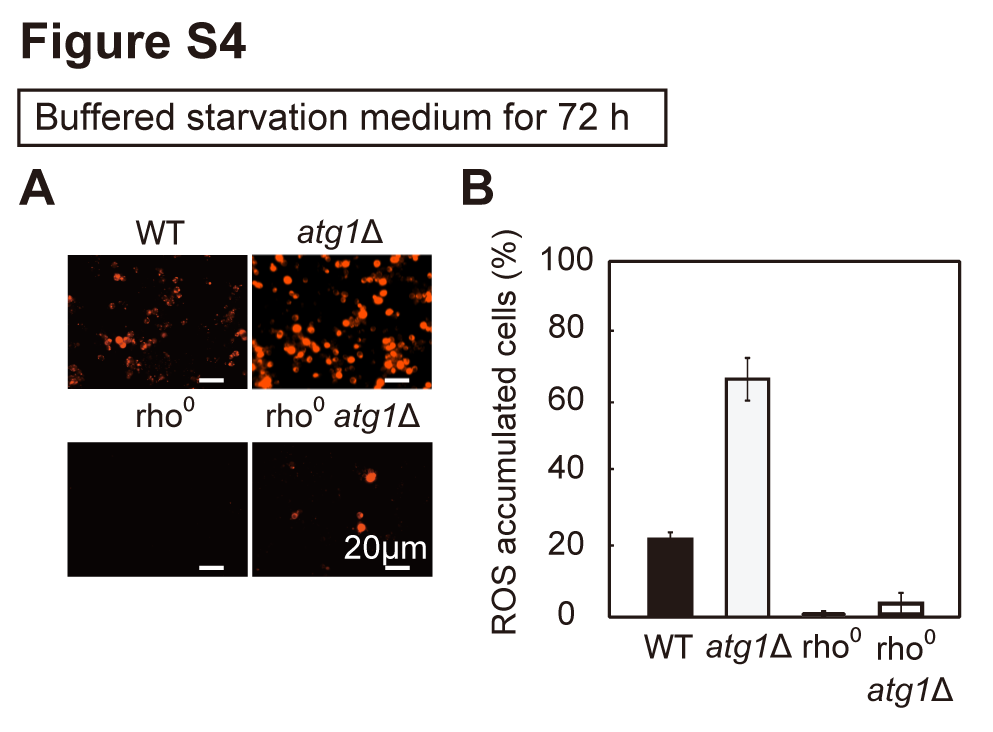

Supplement: Figure S4 — ROS generation in respiratory defective cells. (A–B) WT, atg1Δ, rho0, and rho0 atg1Δ cells were transferred to SD-N +50 mM MES-KOH (pH 6.2) medium for 72 hours. ROS accumulation was detected by DHE staining (A). Each photo contains about 200 cells. Scale bar, 20 µm. (B) Quantification of ROS accumulated cells (n>200 cells). This data represents the average of three independent experiments and bars indicate standard deviations. (TIF) [file pone.0017412.s004.tif]

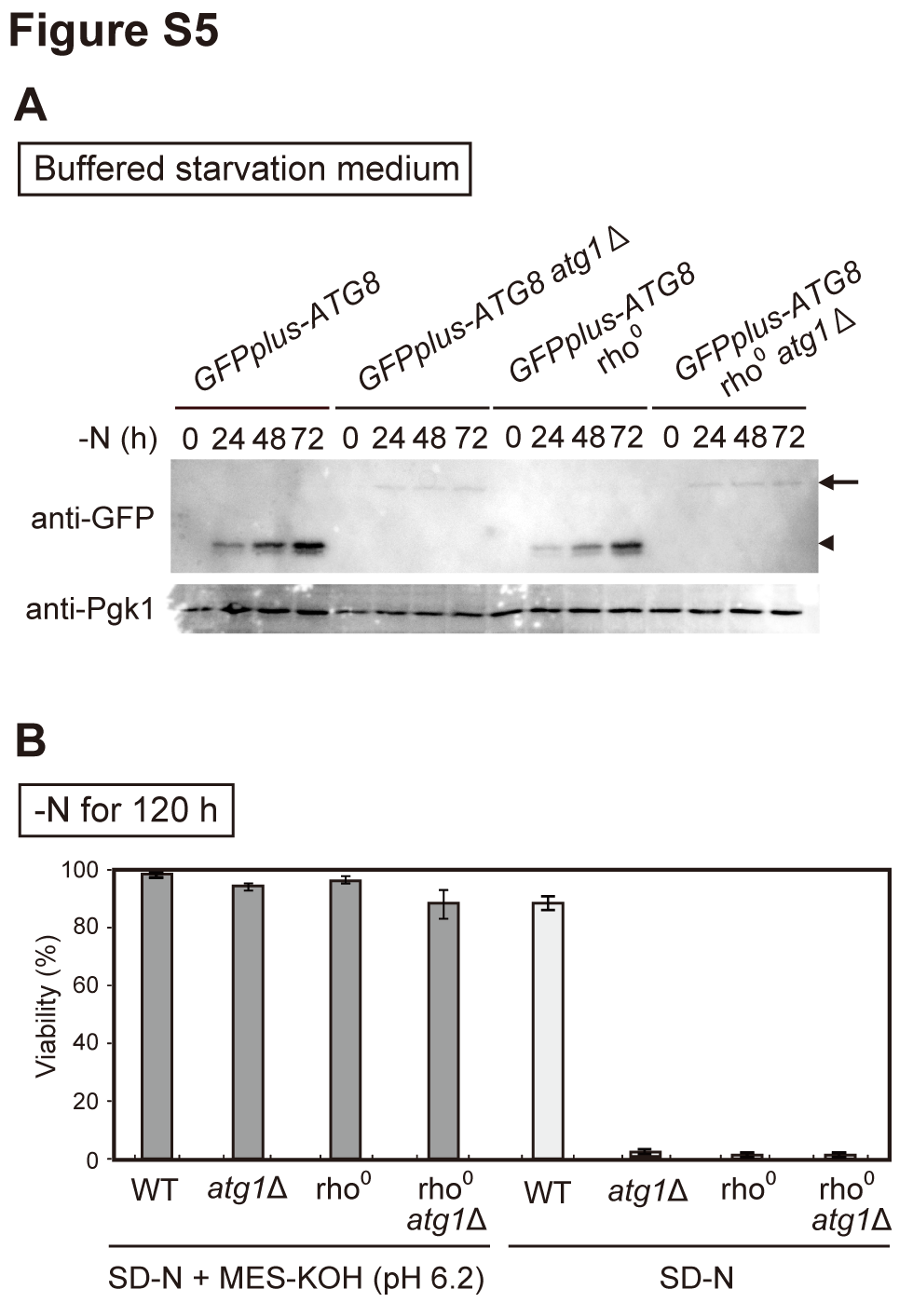

Supplement: Figure S5 — Respiratory deficient cells during nitrogen starvation. (A) WT, atg1Δ, rho0, and rho0 atg1Δ cells expressing GFP-Atg8 were transferred to SD-N +50 mM MES-KOH (pH 6.2) medium for the indicated time. During autophagy process, GFP-Atg8 (depicted by arrow) is delivered to the vacuole, and hydrolyzed to generate free GFP (depicted by arrowhead). Generation of free GFP indicates transport of the marker to the vacuole. Lysates were prepared using a Multi-Beads Shocker (model MB601NIHS, Yasui Kikai Co. Osaka, Japan) and subjected to immunoprecipitation with anti-GFP and anti-Pgk1. Pgk1 was used as loading control. (B) WT, atg1Δ, rho0, and rho0 atg1Δ cells were transferred to SD-N or SD-N +50 mM MES-KOH (pH 6.2). Cell viability was examined by phloxine B. This data represents the average of three independent experiments and bars indicate standard deviations. (TIF) [file pone.0017412.s005.tif]
