# Supplementary material for: Starvation Induced Cell Death in Autophagy-Defective Yeast Mutants Is Caused by Mitochondria Dysfunction
Source: PLoS One. 2011 Feb 25;6(2):e17412. doi: 10.1371/journal.pone.0017412 (PMC3045454; doi:10.1371/journal.pone.0017412)
Supplement: Table S1 — Yeast strains used in this study. (PDF) [file pone.0017412.s006.pdf]

Table S1

Strain	Genotype	Source
**SEY6210**	***MATα leu2 ura3 his3 trp1 lys2 suc2-Δ9***	**(1)**
**SHY2**	**SEY6210; *atg1::kanMX4***	**This study**
**SHY3**	**SEY6210; *atg11::kanMX4***	**This study**
**SHY29**	**SEY6210; *atg8::GFPplus-ATG8:: hphNT1 vph1::VPH1-mCherry::kanMX4***	**This study**
**SHY46**	**SEY6210; *atg8::GFPplus-ATG8:: hphNT1 vph1::VPH1-mCherry::kanMX4 atg1::natNT2***	**This study**
**SHY58**	**SEY6210; *atg7:: natNT2***	**This study**
**SHY70**	**SEY6210; *atg2::kanMX4***	**This study**
**SHY75**	**SEY6210; *atg15::hphNT1***	**This study**
**SHY80**	**SEY6210; *his3Δ1::GPD^{p}-mito-mCherry::CgHIS3***	**This study**
**SHY81**	**SEY6210; *his3Δ1::GPD^{p}-mito-mCherry::CgHIS3***	**This study**
**SHY86**	**SEY6210; *rho^{0}***	**This study**
**SHY94**	**SEY6210; *rho^{0} atg1::kanMX4***	**This study**
**SHY139**	**SEY6210; *cta1::CTA1-3xFLAG::kanMX4***	**This study**
**SHY140**	**SEY6210; *cta1::CTA1-3xFLAG::kanMX4 atg1::natNT2***	**This study**
**SHY141**	**SEY6210; *ctt1::CTT1-3xFLAG::kanMX4***	**This study**
**SHY142**	**SEY6210; *ctt1::CTT1-3xFLAG::kanMX4 atg1::natNT2***	**This study**
**SHY193**	**SEY6210; *rho^{0} atg8::GFPplus-ATG8:: hphNT1 vph1::VPH1-mCherry::kanMX4***	**This study**
**SHY194**	**SEY6210; *rho^{0} atg8::GFPplus-ATG8:: hphNT1 vph1::VPH1-mCherry::kanMX4 atg1::natNT2***	**This study**
**SHY196**	**SEY6210; *atg32::hphNT1***	**This study**

1. Robinson JS, Klionsky DJ, Banta LM, Emr SD (1988) Protein sorting in *Saccharomyces cerevisiae*: isolation of mutants defective in the delivery and processing of multiple vacuolar hydrolases. *Mol Cell Biol* 8: 4936-48.
